# Type II Secretory Phospholipase A_2_ and Prognosis in Patients with Stable Coronary Heart Disease: Mendelian Randomization Study

**DOI:** 10.1371/journal.pone.0022318

**Published:** 2011-07-22

**Authors:** Lutz P. Breitling, Wolfgang Koenig, Marcus Fischer, Ziad Mallat, Christian Hengstenberg, Dietrich Rothenbacher, Hermann Brenner

**Affiliations:** 1 Division C070 Clinical Epidemiology and Aging Research, German Cancer Research Center (DKFZ), Heidelberg, Germany; 2 Department of Internal Medicine II-Cardiology, University of Ulm Medical Center, Ulm, Germany; 3 Klinik und Poliklinik für Innere Medizin II, University of Regensburg, Regensburg, Germany; 4 Division of Cardiovascular Medicine, Addenbrooke's Hospital, Cambridge, United Kingdom; 5 Institute of Epidemiology and Medical Biometry, Ulm University, Ulm, Germany; Innsbruck Medical University, Austria

## Abstract

**Background:**

Serum type II secretory phospholipase A_2_ (sPLA_2_-IIa) has been found to be predictive of adverse outcomes in patients with stable coronary heart disease. Compounds targeting sPLA_2_-IIa are already under development. This study investigated if an association of sPLA_2_-IIa with secondary cardiovascular disease (CVD) events may be of causal nature or mainly a matter of confounding by correlated cardiovascular risk markers.

**Methodology/Principal Findings:**

Eight-year follow-up data of a prospective cohort study (KAROLA) of patients who underwent in-patient rehabilitation after an acute cardiovascular event were analysed. Associations of polymorphisms (SNP) in the sPLA_2_-IIa-coding gene *PLA2G2A* with serum sPLA_2_-IIa and secondary fatal or non-fatal CVD events were examined by multiple regression. Hazard ratios (HR) were compared with those expected if the association between sPLA_2_-IIa and CVD were causal. The strongest determinants of sPLA_2_-IIa (rs4744 and rs10732279) were associated with an increase of serum concentrations by 81% and 73% per variant allele. HRs (95% confidence intervals) estimating the associations of the SNPs with secondary CVD events were increased, but not statistically significant (1.16 [0.89–1.51] and 1.18 [0.91–1.52] per variant allele, respectively). However, these estimates were very similar to those expected when assuming causality (1.18 and 1.17), based on an association of natural log-transformed sPLA_2_-IIa concentration with secondary events with HR = 1.33 per unit.

**Conclusion:**

The present findings regarding genetic polymorphisms, determination of serum sPLA_2_-IIa, and prognosis in CVD patients are consistent with a genuine causal relationship and thus might point to a valid drug target for prevention of secondary CVD events.

## Introduction

Recent studies have demonstrated that the pro-inflammatory enzyme type II secretory phospholipase A_2_ (sPLA_2_-IIa) provides prognostic information beyond other established risk markers in both primary [1], [2] and secondary [3], [4], [5] cardiovascular disease events. Further elucidating the causality of these associations would be of outstanding and immediate relevance, as therapeutic agents targeting sPLA_2_ are already under development [6], [7]. However, since cardiovascular risk factors tend to be closely correlated, it remains unclear whether the associations reported to date reflect genuine causality, or are spurious and due to imperfect adjustment for confounding variables especially in observational studies.

As reviewed in detail elsewhere [8], [9], [10], several findings from experimental studies would be consistent with a causal role of this enzyme in atherosclerosis and cardiovascular disease. For example, modifications by sPLA_2_-IIa contribute to the affinity of low-density lipoproteins to extracellular proteoglycans, which can ultimately result in higher extracellular lipoprotein accumulation, an important component of atherogenesis [8]. Overexpression experiments have further shown that macrophage sPLA_2_-IIa contributes to oxidative stress and plays a role in foam cell growth as well as in the development of atherosclerotic lesions at least in mice [9], [11].

Mendelian randomization is an approach in which the magnitude of the presumably unconfounded association between genetic determinants of a risk marker and an outcome of interest is employed to make inferences regarding the extent of causal association between the marker and the disease [12], [13], [14]. If, for instance, a genetic variant is associated with a doubling in marker concentrations, and a doubling of marker concentrations is associated with an x-fold elevation in risk, the genetic variant should likewise be associated with an x-fold risk increase, but only if the marker–disease association is not due to confounding. Otherwise, the genetic variant–disease association should be weakened or even nil, since—similar to randomized intervention allocation in clinical trials—the randomized allocation of alleles during meiosis essentially ensures that no association exists between the genetic variant and the confounders (known or unknown) commonly distorting the marker–disease association.

In the present work, we investigated the association between single nucleotide polymorphisms (SNP) in the coding gene *PLA2G2A* and serum levels of sPLA_2_-IIa in a large cohort of patients with stable coronary heart disease featuring eight years of follow-up. Drawing upon the idea of Mendelian randomization, we subsequently aimed to elucidate whether the previously described association between sPLA_2_-IIa and secondary CVD events was causal or predominantly due to confounding.

## Methods

### Ethics Statement

The study protocol was approved by the ethics committees of the physicians' chambers of Hessen and Baden-Württemberg, and of the Universities of Ulm and Heidelberg. All study participants gave written informed consent.

### Study Design

Details of the study design have been reported previously [15]. In brief, patients participating in an in-patient rehabilitation programme after experiencing an acute cardiovascular event (acute coronary syndrome, myocardial infarction, coronary revascularization) were recruited in two specialized rehabilitation clinics in the South and West of Germany from January 1999 to May 2000. Only subjects aged 30–70 years admitted within three months after their acute event were included in the study.

Baseline data were obtained by standardized self-administered questionnaires at the beginning of in-patient rehabilitation, and complemented by extracting relevant data from hospital records, including secondary diagnoses and drug prescriptions at discharge from the rehabilitation programme. Active follow-up by mailed standardized questionnaires was carried out after 1, 3, 4.5, 6 and 8 years. Information on secondary cardiovascular events (myocardial infarction and stroke) was provided by the general practitioners of the participants. If participants were deceased, de-facto death certificates including the major cause of death were obtained from Public Health authorities.

### Laboratory Measurements

A fasting blood sample was taken at the end of the in-patient rehabilitation and stored at −80°C until analysis. Serum concentrations of sPLA_2_-IIa mass were determined using a commercially available ELISA assay (Cayman Chemical Co., Ann Arbor, MI/USA) with a lower detection limit of about 0.3 ng/mL. In addition, sPLA_2_-IIa activity was determined using a selective fluorometric assay [5].

In an attempt to cover the genetic variation in the *PLA2G2A* gene coding for sPLA_2_-IIa, we selected validated SNPs with minor allele frequencies above 5% for which working TaqMan assays were available (rs876018, rs955587, rs4744, rs10732279, rs3753827, rs11573156). Intergenic SNPs were chosen from nearby well conserved regions (rs10799599, rs10916685, rs818678). Genotyping was done using 5′ exonuclease TaqMan® technology (Applied Biosystems, Foster City, CA, USA). For each genotyping experiment 10 ng DNA was employed in a total volume of 5 µl containing 1x TaqMan® Genotyping Master Mix (Applied Biosystems). PCR reaction with pre-designed TaqMan genotyping assays (Applied Biosystems) and post-PCR endpoint plate read was carried out according to the manufacturer's instructions on a Applied Biosystems 7900HT Real-Time PCR System. Sequence Detection System software version 2.3 (Applied Biosystems) was used to assign genotypes applying the allelic discrimination test. DNA was genotyped with duplicates of samples (20%) to assess genotype quality. No genotyping discrepancies were detected. After checking for deviations from Hardy-Weinberg equilibrium, haplotypes were estimated using the software PHASE version 2.1.1 [16], [17]. Linkage disequilibrium plots were created using Haploview version 4 [18].

### Statistical Analysis

Linear regression models adjusted for age and sex with the natural logarithm (*ln*)-transformed sPLA_2_-IIa mass concentration or activity as the dependent variable were used to estimate the association of individual SNP genotypes with serum sPLA_2_-IIa. Multi-locus models adjusted for age and sex were constructed by stepwise selection from all SNPs in Hardy-Weinberg equilibrium, using permissive entry and stay significance levels of *P* = 0.4 and *P* = 0.6. The final model was then chosen as the one with the most favourable predicted residual sum of squares from 10-fold cross-validation (SAS procedure GLMSELECT [19]). The association of genotypes with secondary CVD events (fatal or non-fatal myocardial infarction or stroke) was assessed using Cox proportional hazards models, likewise adjusted for age and sex. For subjects free of secondary CVD events at follow-up, the survival time was censored if treating practitioners could not be contacted anymore.

Potential confounders of the genotype–disease relationship included the following covariables: sex, age, smoking status, diagnosis of acute myocardial infarction, diabetes, hypertension, number of vessels affected, discharge prescriptions of beta-blocking agents, ACE inhibitors, diuretics, lipid-lowering drugs and ASS, body mass index, HDL-, LDL- and total cholesterol, triglycerides, Lp-PLA_2_, interleukin-6, hsCRP, NT-proBNP, RBP-4, cystatin C, adiponectin, and creatinine clearance. Testing was done by Kruskal-Wallis (continuous variables) or χ^2^ test (categorical variables).

The updated Cox proportional hazards regression model estimating the association between sPLA_2_-IIa concentration or activity and secondary cardiovascular events was adjusted for all variables identified as important confounders or covariables in previous detailed analyses based on 4-year follow-up of our cohort [5], i.e. age (continuous), sex, smoking status, diagnosis of diabetes and hypertension, study site, type of acute intervention, baseline HDL- and LDL-cholesterol (continuous), discharge prescription of lipid-lowering drugs, body mass index (continuous), and number of affected vessels.

#### Mendelian Randomization Approach

As both the genotype–marker (regression estimate *β*_g→m_) and marker–disease associations (regression estimate *β*_m→d_, hazard ratio  =  exp(*β*_m→d_)) were modelled based on *ln*-transformed sPLA_2_-IIa concentrations, the expected association between genotypes and disease under the assumption of no confounding could be directly calculated as *β*_g→d_ = *β*_g→m_×*β*_m→d_ (expected hazard ratio  =  exp(*β*_g→d_)). Confidence intervals for the expected hazard ratios were obtained as described elsewhere [20]: assuming the regression estimates to originate from normal distributions around their point estimate and with their respective standard deviation, 1,000,000 combinations of *β*_g→m_ and *β*_m→d_ were drawn pseudo-randomly, and the 2.5th and 97.5th percentiles of the resulting 1,000,000 hazard ratios were taken as empirical estimates of the 95% confidence interval limits. Note that the Mendelian randomization approach was restricted to sPLA_2_-IIa mass concentrations, as the genetic determination of activity was somewhat less pronounced.

Statistical analyses were conducted using SAS [19]; the empirical 95% confidence interval of the expected hazard ratios was obtained using R [21]. Tests were two-sided with alpha  = 0.05.

## Results

The nine SNPs could be genotyped in 991 to 1,014 of 1,019 participants for whom follow-up information for the combined outcome of fatal and non-fatal secondary CVD events was also available (84.5% of 1,206 subjects originally included; in each follow-up round, response rates for both patient and general practitioner questionnaires were ≥93%). The subjects in the present analysis set had a median (interquartile range) age of 61 (54–65) years and BMI of 26.6 (24.8–28.7) kg/m^2^. The study population included 85.1% males, and 58.3% had suffered an acute myocardial infarction. Further baseline characteristics along with sPLA_2_-IIa concentrations and activities are reported in Table 1. The blood draw for sPLA_2_-IIa measurements took place on average±standard deviation 43±13 days after the acute event or procedure in the acute care hospital, and sPLA_2_-IIa concentration as well as activity showed a weak negative correlation with the time between the event/procedure and the blood draw (Spearman coefficients of −0.11 and −0.12, respectively). As the time between the event/procedure and the blood draw, which ranged from 16 to 110 days, was neither significantly associated with any of the genotypes, nor with the risk of secondary cardiovascular disease events, this variable was not further considered or controlled for in the subsequent analyses.

**Table 1:** Baseline characteristics and sPLA2-IIa concentrations and activities in patients with stable coronary heart disease.

				sPLA2-IIa concentration (ng/mL)		sPLA2-IIa activity (nmol/mL/min)	
Participant characteristic		N	%	Median	Inter-quartile range	Median	Inter-quartile range
Overall population^a^		1012	100	2.50	1.45–4.86	1.21	0.93–1.58
Age at baseline	30–39 years	20	2	1.47	0.92–1.91	0.95	0.76–1.22
	40–49 years	125	12	1.71	1.10–3.51	1.07	0.89–1.41
	50–59 years	292	29	2.08	1.31–3.91	1.12	0.87–1.44
	60–70 years	575	57	2.99	1.71–5.63	1.30	0.97–1.70
Sex	Female	152	15	4.99	2.20–8.21	1.53	1.17–2.15
	Male	860	85	2.33	1.39–4.20	1.16	0.91–1.51
Patient group	Acute coronary syndrome	127	13	2.00	1.26–3.81	1.11	0.93–1.39
	Coronary intervention	296	29	2.77	1.69–5.36	1.25	0.94–1.68
	Myocardial infarction	589	58	2.48	1.40–4.68	1.21	0.92–1.57
Body mass index	≤25 kg/m^2^	285	28	2.47	1.44–4.99	1.16	0.92–1.53
	>25–30 kg/m^2^	569	56	2.41	1.44–4.61	1.21	0.91–1.55
	>30 kg/m^2^	157	16	3.14	1.49–5.98	1.35	1.01–1.79
Disease history	Diabetes mellitus	177	18	3.45	1.80–6.26	1.35	1.05–1.76
	Hypertension	564	56	2.77	1.54–5.47	1.26	0.94–1.67
Discharge prescriptions	ACE-inhibitor	535	53	2.63	1.46–4.81	1.22	0.90–1.60
	Lipid-lowering drugs	780	77	2.33	1.41–4.47	1.19	0.92–1.53
Smoking history	Never	316	31	2.51	1.48–5.49	1.24	0.94–1.66
	Formerly	645	64	2.48	1.42–4.63	1.20	0.92–1.56
	Currently	51	5	2.66	1.73–5.09	1.16	0.95–1.41

a N and percentages refer to the subjects with concentration measurements available. Activity measurements were missing for 22 of these.

### Genetic Determinants of sPLA_2_-IIa Concentration and Activity

**Figure 1:**
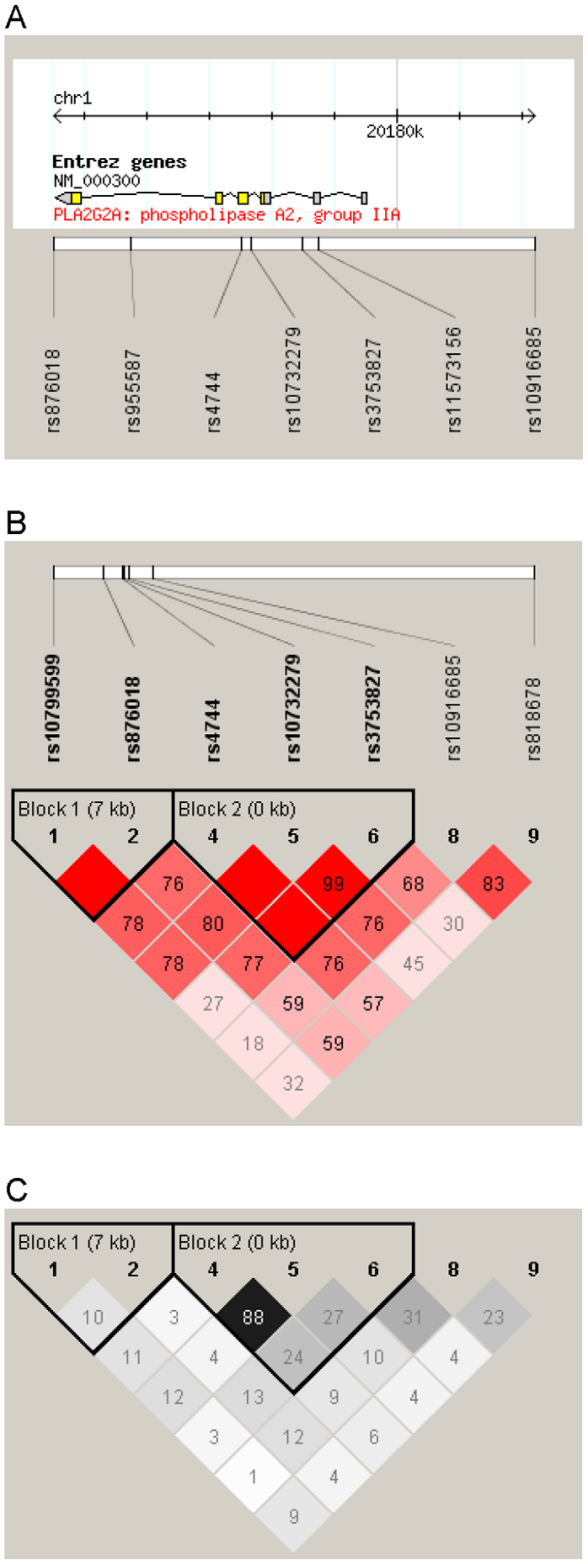
Location of the single nucleotide polymorphisms genotyped in this study in relation to the *PLA2G2A* gene (Panel A) and linkage disequilibrium patterns between the markers considered for haplotype estimation (Panels B and C). Panel A: depicted is chromosome 1, 20174.5–20182.2 kb; not shown are rs10799599 at position 20167087 and rs818678 at position 20238917. Panels B and C: D' values in Haploview standard colouring scheme are shown in B, r^2^ values in Haploview r^2^ colouring scheme are shown in C. Haplotype blocks were identified by Haploview's confidence interval option.

The location of the genotyped SNPs relative to *PLA2G2A* is depicted in Figure 1, along with linkage disequilibrium plots. Minor allele frequencies generally in line with dbSNP information were observed for all loci (Table 2). As can be seen in the table, two SNPs mildly deviated from Hardy-Weinberg equilibrium. These were excluded from haplotype and survival analyses. As demonstrated in Table 2, most of the SNPs were strongly associated with sPLA_2_-IIa concentrations. Analysing *ln*-transformed concentrations, which were approximately normally distributed, the proportion of variance explained by individual SNPs reached up to 16%. The strongest effect was observed in rs4744 variant homozygotes, in whom sPLA_2_-IIa was increased by almost 200% in comparison to wildtype homozygotes. Two- and three-loci haplotypes constructed from the block1 and block2 SNPs (Figure 1B and 1C), respectively, were also significantly associated with plasma sPLA_2_-IIa mass, but effect sizes were lower than for rs4744 and rs10732279 individually (not shown). When instead employing stepwise model selection and cross-validation, the final model included rs4744 and rs10799599 (regardless of whether additive or three-categorical genotype effects were assumed), suggesting that these variants might exert independent effects on sPLA_2_-IIa concentrations. The regression coefficients in the additive final model were *β* = 0.544 for rs4744 and *β* = –0.120 for rs10799599.

**Table 2:** Genetic determination of sPLA_2_-IIa concentrations.

						Genotypic model (three-categorical)				Additive model	
Single nucleotide polymorphism (n)	Common/ rare allele	Minor allele frequency	*P*_χ^2^(HWE)_	R^2^	P*^a^	Δ%^b^	(95% CI)	Δ%^c^	(95% CI)	Δ%^d^	(95% CI)
rs10799599 (995)	C/G	0.37	0.23	0.049	<0.0001	−20.3	(−28.6 to −11.0)	−44.8	(−53.2 to −34.8)	−24.2	(−29.8 to −18.2)
rs876018 (989)	A/T	0.15	0.52	0.008	0.047	−15.6	(−25.3 to −4.5)	2.5	(−26.7 to 43.3)	−10.3	(−19.1 to −0.5)
rs955587 (994)	C/T	0.12	0.01	0.007	0.0095	−8.3	(−19.2 to 4.2)	−44.1	(−71.9 to 11.2)	−10.3	(−20.5 to 1.2)
rs4744 (996)	G/A	0.23	0.21	0.158	<0.0001	87.6	(69.4 to 107.8)	199.8	(138.1 to 277.6)	81.3	(66.9 to 96.9)
rs10732279 (1008)	T/C	0.26	0.33	0.148	<0.0001	77.2	(60.1 to 96.2)	186.5	(133.3 to 251.8)	73.3	(60.0 to 87.6)
rs3753827 (1007)	C/A	0.44	0.83	0.033	<0.0001	−24.7	(−33.1 to −15.2)	−36.1	(−45.0 to −25.7)	−20.6	(−26.3 to −14.5)
rs11573156 (1001)	G/C	0.25	0.04	0.160	<0.0001	90.0	(71.8 to 110.1)	188.1	(129.3 to 262.0)	81.1	(66.8 to 96.6)
rs10916685 (995)	A/T	0.34	0.46	0.015	0.0015	−17.1	(−25.9 to −7.2)	−27.1	(−38.6 to −13.5)	−15.4	(−21.7 to −8.5)
rs818678 (984)	C/T	0.39	0.88	0.013	0.0002	−12.0	(−21.6 to −1.3)	−26.1	(−37.0 to −13.3)	−13.6	(−19.9 to −6.8)

a Based on non-parametric Kruskal-Wallis test on untransformed sPLA_2_-IIa concentrations.

b Percent change in geometric mean sPLA_2_-IIa concentration in heterozygotes in reference to common homozygotes.

c Percent change in geometric mean sPLA_2_-IIa concentration in rare homozygotes in reference to common homozygotes.

d Percent change in geometric mean sPLA_2_-IIa concentration per minor allele present.

Genotype distributions, proportion of variance in *ln*-transformed sPLA_2_-IIa serum concentration explained by the SNPs studied (R^2^; one-way ANOVA), and results from age- and sex-adjusted regression models predicting *ln*-transformed sPLA_2_-IIa concentrations from genotypes.

Table 3 shows the analoguous results for sPLA_2_-IIa activity. Whereas there likewise were strongly significant assocations with several of the SNPs, the explained variance and %-changes associated with the genetic variants tended to be smaller than in the case of the sPLA_2_-IIa concentrations. The final model based on stepwise model selection and cross-validation included rs4744 and rs10799599 in the three-categorical approach, and additionally rs10732279 and rs818678 in the additive approach (*β*_rs4744_ = 0.115, *β*_rs10799599_ = –0.062, *β*_rs10732279_ = 0.091, *β*_rs818678_ = –0.041).

**Table 3:** Genetic determination of sPLA_2_-IIa activities.

			Genotypic model (three-categorical)				Additive model	
Single nucleotide polymorphism (n)	R^2^	*P* a	Δ%^b^	(95% CI)	Δ%^c^	(95% CI)	Δ%^d^	(95% CI)
rs10799599 (974)	0.046	<0.0001	−11.7	(−16.6 to −6.49)	−24.5	(−30.8 to −17.7)	−12.7	(−16.1 to −9.17)
rs876018 (967)	0.003	0.16	−2.5	(−8.50 to 3.80)	13.1	(−5.21 to 34.9)	0.2	(−4.99 to 5.75)
rs955587 (973)	0.011	0.0008	−5.8	(−11.7 to 0.57)	−32.6	(−52.4 to −4.40)	−7.2	(−12.8 to −1.27)
rs4744 (974)	0.109	<0.0001	28.1	(21.3 to 35.2)	61.9	(43.2 to 83.0)	27.7	(22.2 to 33.5)
rs10732279 (986)	0.105	<0.0001	24.8	(18.3 to 31.7)	60.7	(44.2 to 79.0)	25.8	(20.6 to 31.1)
rs3753827 (985)	0.020	<0.0001	−11.9	(−17.1 to −6.34)	−14.0	(−20.5 to −7.04)	−7.9	(−11.4 to −4.29)
rs11573156 (979)	0.110	<0.0001	28.2	(21.5 to 35.2)	61.0	(42.8 to 81.5)	27.6	(22.2 to 33.3)
rs10916685 (973)	0.003	0.21	−4.5	(−9.86 to 1.26)	−5.2	(−13.2 to 3.58)	−3.2	(−7.01 to 0.77)
rs818678 (963)	0.021	<0.0001	−7.3	(−12.6 to −1.63)	−17.7	(−24.2 to −10.6)	−8.8	(−12.3 to −5.19)

a Based on non-parametric Kruskal-Wallis test on untransformed sPLA2-II activities.

b Percent change in geometric mean sPLA2-II activity in heterozygotes in reference to common homozygotes.

c Percent change in geometric mean sPLA2-II activity in rare homozygotes in reference to common homozygotes.

d Percent change in geometric mean sPLA2-II activity per minor allele present.

Proportion of variance in *ln*-transformed sPLA_2_-IIa serum activity explained by the SNPs studied (R^2^; one-way ANOVA), and results from age- and sex-adjusted regression models predicting *ln*-transformed sPLA_2_-IIa activities from genotypes.

There were no pronounced associations between the SNP genotypes and any of the variables screened as potential confounders (details not shown). Only 4 tests yielded p-values below 0.05, namely for the associations between rs955587 and age (Kruskal-Wallis *P* = 0.028), rs3753827 and HDL-cholesterol (Kruskal-Wallis *P* = 0.018), and between rs818678 and HDL-cholesterol (Kruskal-Wallis *P* = 0.0033) and prescription of diuretics (χ^2^ *P* = 0.014). Note, that 9 SNPs were tested against 25 variables, i.e. 225 tests were considered.

### Genetic Variants, sPLA_2_-IIa, and Secondary CVD Events

Until year 8 follow-up, 149 secondary cardiovascular events occurred in the analysis population, including 49 and 41 non-fatal myocardial infarctions and strokes, and 59 deaths with cardiovascular major cause. The genotyped individuals contributed a total of 6889.4 person-years with a median follow-up time of 8.1 years. In Cox regression analysis adjusting for multiple covariates as included in the 4-year follow-up model [5], a unit increase in *ln*-transformed sPLA_2_-IIa concentrations was associated with an elevated risk of adverse outcomes with a hazard ratio (HR; 95% confidence interval) of 1.33 (1.09–1.63; *P* = 0.0057). Additionally exploring the association of the *ln*-transformed concentrations with all-cause mortality (total number of deaths observed: 116) revealed no significant association (HR = 1.12 [0.89–1.41]). The results for *ln*-transformed sPLA_2_-IIa activity were not statistically significant (secondary cardiovascular events: HR = 1.41 [0.94–2.11]; all-cause mortality: HR = 0.98 [0.64–1.53]).

Observed associations between *PLA2G2A* SNPs and secondary cardiovascular events estimated by Cox proportional hazards regression are shown in the upper part of Table 4, both based on genotypic and additive codings of the SNPs. The confidence intervals around the estimated SNP effects all included the Null value of no association (HR = 1). However, for the SNPs most strongly associated with sPLA_2_-IIa concentrations, the hazard ratios observed were intriguingly consistent with those expected assuming a causal nature of the sPLA_2_-IIa–disease association (bottom of Table 4). This became particularly clear for the statistically more stable additive models which are less affected by fluctuations due to small stratum sizes. Using a genotype score constructed from the two SNPs in the sPLA_2_-IIa concentration stepwise selection regression model (score = number of rs4744 variant alleles + (−0.120/0.544)× number of rs10799599 variant alleles) as a predictor of secondary cardiovascular events yielded similar results (HR = 1.16 [0.91–1.47]; expected HR = 1.17 [1.05–1.31]).

**Table 4:** Observed and expected relative risks.

	Genotype		HR^a^	(95% CI)	HR^b^	(95% CI)
Observed associations	rs10799599	CC	1	ref.	1	ref.
		CG	0.88	(0.62–1.23)	0.85	(0.67–1.09)
		GG	0.71	(0.40–1.24)		
	rs876018	AA	1	ref.	1	ref.
		AT	1.24	(0.87–1.78)	1.11	(0.82–1.50)
		TT	0.76	(0.24–2.40)		
	rs4744	GG	1	ref.	1	ref.
		GA	1.09	(0.77–1.53)	1.16	(0.89–1.51)
		AA	1.52	(0.79–2.93)		
	rs10732279	TT	1	ref.	1	ref.
		TC	1.04	(0.74–1.46)	1.18	(0.91–1.52)
		CC	1.65	(0.94–2.93)		
	rs3753827	CC	1	ref.	1	ref.
		CA	0.71	(0.50–1.02)	0.89	(0.71–1.13)
		AA	0.87	(0.56–1.35)		
	rs10916685	AA	1	ref.	1	ref.
		AT	0.83	(0.59–1.17)	0.91	(0.72–1.16)
		TT	0.92	(0.55–1.54)		
	rs818678	CC	1	ref.	1	ref.
		CT	1.03	(0.72–1.47)	1.03	(0.81–1.30)
		TT	1.06	(0.65–1.74)		
Expected associations	rs4744	GG	1	ref.	1	ref.
		GA	1.20	(1.05–1.37)	1.18	(1.05–1.34)
		AA	1.37	(1.09–1.74)		
	rs10732279	TT	1	ref.	1	ref.
		TC	1.18	(1.05–1.33)	1.17	(1.05–1.31)
		CC	1.35	(1.09–1.70)		

a Genotypic model (three-categorical), adjusted for age and sex.

b Additive genotype model, adjusted for age and sex.

Observed associations of individual SNPs with secondary cardiovascular events (hazard ratios [HR] from Cox regression), and effects expected by Mendelian randomization if the fully adjusted *ln*[sPLA_2_-IIa] association of HR (95% CI)  = 1.33 (1.09–1.63) is not due to confounding.

## Discussion

In the present study of patients with stable coronary heart disease at baseline, we found a strong determination of serum sPLA_2_-IIa by *PLA2G2A* polymorphisms. Although the associations of these polymorphisms with prognosis of eight years of follow-up were not statistically significant, considerations along the lines of Mendelian randomization suggested that the observations were consistent with a causal association between sPLA_2_-IIa and cardiovascular prognosis and made confounding factors a rather unlikely cause for these patterns.

### Genetic Determination of sPLA_2_-IIa

Pronounced associations of SNPs in *PLA2G2A* with concentrations of sPLA_2_-IIa have been reported previously [22]. As these data had not been published during the planning phase/SNP assay preparation of the present study, our SNP selection was not informed by this previous report. However, there was an overlap of three SNPs, for which results were consistent in both studies (rs876018, rs3753827, rs11573156). Wootton et al. [22] interpreted their haplotype analysis by suggesting that additional loci not captured by their assays would also contribute importantly to sPLA_2_-IIa concentrations. They identified two predominant tagging-SNPs, and it appears noteworthy that the one (rs11573156) located closer to the strongest individual predictors in the present analysis (rs4744 and rs10732279) was not assigned to the same haplotype block as these two SNPs, which had not been covered by Wootton et al. We had excluded rs11573156 due to rather limited deviation from Hardy-Weinberg equilibrium distribution, regardless of which rs4744 and rs10732279 should be considered as relevant candidates for causal loci in future studies of genetic sPLA_2_-IIa determination. The multi-locus model selection approach additionally highlighted rs10799599 as a potential independent predictor of sPLA_2_-IIa activity in our study. Importantly, the strong genetic determination of sPLA_2_-IIa concentrations could furthermore be extended to sPLA_2_-IIa activity, for which the genetic determination was found to be only slightly less pronounced, and possibly somewhat more complex as suggested by the results of the multi-locus models.

### Mendelian Randomization Interpretation

Given the moderate number of secondary CVD events available for the present analyses (137 to 149 depending on the model), the lack of statistical significance in survival analyses was not overly surprising. However, our findings appear qualitatively very different from the rather disappointing results recently obtained by Mendelian randomization approaches pertaining to the role of C-reactive protein in the causation of coronary heart disease [23], [24], [25] or diabetes [26]. For both diseases entities, robust evidence for independent and prospective associations with C-reactive protein had been accumulated through multiple observational studies (details in [25], [26]). In subsequent Mendelian randomization analyses, however, a causal relationship seemed very unlikely. For example, with expected odds ratios (95% confidence interval) of 1.20 (1.07–1.38) [25] and 0.94 (0.94–0.95) [24], the observed odds ratios were 1.01 (0.74–1.38) and 1.00 (0.97–1.02), respectively. Whereas the difference in effect directions in the two cited analyses is due to different codings, the general pattern notably is the same, despite the second study including many more individuals and yielding substantially narrower confidence intervals. In contrast, the patterns in our study appeared to resemble more the evidence presented e.g. for homocysteine with stroke [20]. The starting point for Mendelian randomization analysis in the case of homocysteine was rather similar to the C-reactive protein associations mentioned above. However, the expected odds ratio of 1.20 (1.10–1.31) was approximated fairly well by the meta-analysis of the genotype–disease association, which produced an estimate of 1.26 (1.14–1.40). Mixed results in trials of homocysteine-lowering interventions, of course, are a reminder of the potential difficulties in translating such findings into effective treatments [27].

The homocysteine Mendelian randomization study was based on pooled analyses of more than 10,000 participants [20], and the C-reactive protein analyses estimated associations using data from more than 100,000 subjects [24]. Such large-scale meta-analytic approaches clearly would be preferable for drawing more definite conclusions regarding the causality of sPLA_2_-IIa with respect to prognosis in coronary heart disease patients. Unfortunately, the absolute scarcity of data pertinent to the prognostic value of genetic determinants of sPLA_2_-IIa levels at present is prohibitive in this regard. Also, the prognostic value of sPLA_2_-IIa itself has been investigated only by few studies to date, which, as has been discussed previously [5], might have been biased towards enlarged effect estimates due to small sample sizes.

### Limitations of the Present Work

Apart from moderate statistical power, aggravated by some loss-to-follow-up hardly avoidable in observational epidemiological studies, another limitation of our study was the possibility of participant self-selection due to voluntary study participation. In addition, our study design with recruitment upon arrival in the rehabilitation clinic precluded the analysis of early mortality; nevertheless, the study population represents the group that would benefit from secondary prevention means. As associations and causal relationships could plausibly differ between the early/acute and late/chronic disease phases, the former should not be considered the subject of the KAROLA study. Altogether, these issues should not affect the internal validity of our findings, and our analysis population indeed can still be considered representative for a substantial proportion of patients with stable coronary heart disease. Finally, results from the present work were based on a mostly male study population, and sex-specific aspects of the relationships described need to be addressed in additional cohorts.

The absence of confounding of the genetic polymorphism–disease association is one of the assumptions for valid Mendelian randomization analyses [12]. While this usually is ensured due to the random allocation of alleles during meiosis, which lies at the core of the Mendelian randomization concept, the situation might be somewhat different in studies of secondary CVD event, where genotypes associated with higher risk for primary manifestations may introduce some selection bias and compromise the naturally occurring randomization. However, when we examined associations between the SNPs and a multitude of variables that have previously been reported to be associated with cardiovascular risk, only four *P*-values were smaller than 0.05. Given the multiplicity of testing in this part of the analysis, these results made it unlikely that the SNPs–secondary CVD events relationship was distorted to any relevant degree by confounding due to known risk factors.

### Conclusions and Perspective

Confirmatory studies are clearly needed to corroborate our results. However, our findings are consistent with the prospect of sPLA_2_-IIa being a ‘chicken’ rather than just another ‘egg’ with respect to cardiovascular disease, very much in contrast to C-reactive protein [28]. Given the global burden of morbidity and mortality imposed by cardiovascular disease, such hints to plausible causal relationships are of utmost importance and of immediate relevance to future research priorities. Whereas the investigation of clinical interventions with existing or yet-to-be-developed sPLA_2_-IIa-inhibiting drugs [6], [7] will necessarily be the realm of randomized clinical trials, the present results strengthen the pathophysiological rationale for conducting such studies. In particular given the disappointing tale of causality and Mendelian randomization of C-reactive protein, it furthermore appears reasonable to include the most important genetic determinants of sPLA_2_-IIa in any forthcoming studies of the prognostic value of this marker, in order to capitalize on Mendelian randomization from the beginning on.

## References

[1] Boekholdt SM, Keller TT, Wareham NJ, Luben R, Bingham SA (2005). Serum levels of type II secretory phospholipase A2 and the risk of future coronary artery disease in apparently healthy men and women: the EPIC-Norfolk Prospective Population Study.. Arterioscler Thromb Vasc Biol.

[2] Mallat Z, Benessiano J, Simon T, Ederhy S, Sebella-Arguelles C (2007). Circulating secretory phospholipase A2 activity and risk of incident coronary events in healthy men and women: the EPIC-Norfolk study.. Arterioscler Thromb Vasc Biol.

[3] Kugiyama K, Ota Y, Takazoe K, Moriyama Y, Kawano H (1999). Circulating levels of secretory type II phospholipase A(2) predict coronary events in patients with coronary artery disease.. Circulation.

[4] Mallat Z, Steg PG, Benessiano J, Tanguy ML, Fox KA (2005). Circulating secretory phospholipase A2 activity predicts recurrent events in patients with severe acute coronary syndromes.. J Am Coll Cardiol.

[5] Koenig W, Vossen CY, Mallat Z, Brenner H, Benessiano J (2009). Association between type II secretory phospholipase A2 plasma concentrations and activity and cardiovascular events in patients with coronary heart disease.. Eur Heart J.

[6] Koenig W, Khuseyinova N (2009). Lipoprotein-associated and secretory phospholipase A2 in cardiovascular disease: the epidemiological evidence.. Cardiovasc Drugs Ther.

[7] Rosenson RS (2009). Future role for selective phospholipase A2 inhibitors in the prevention of atherosclerotic cardiovascular disease.. Cardiovasc Drugs Ther.

[8] Jonsson-Rylander AC, Lundin S, Rosengren B, Pettersson C, Hurt-Camejo E (2008). Role of secretory phospholipases in atherogenesis.. Curr Atheroscler Rep.

[9] Rosenson RS, Gelb MH (2009). Secretory phospholipase A2: a multifaceted family of proatherogenic enzymes.. Curr Cardiol Rep.

[10] Mallat Z, Lambeau G, Tedgui A (2010). Lipoprotein-associated and secreted phospholipases A in cardiovascular disease: roles as biological effectors and biomarkers.. Circulation.

[11] Tietge UJ, Pratico D, Ding T, Funk CD, Hildebrand RB (2005). Macrophage-specific expression of group IIA sPLA2 results in accelerated atherogenesis by increasing oxidative stress.. J Lipid Res.

[12] Sheehan NA, Didelez V, Burton PR, Tobin MD (2008). Mendelian randomisation and causal inference in observational epidemiology.. PLoS Med.

[13] Davey Smith G, Ebrahim S (2003). ‘Mendelian randomization’: can genetic epidemiology contribute to understanding environmental determinants of disease?. Int J Epidemiol.

[14] Davey Smith G, Ebrahim S (2004). Mendelian randomization: prospects, potentials, and limitations.. Int J Epidemiol.

[15] Rothenbacher D, Koenig W, Brenner H (2006). Comparison of N-terminal pro-B-natriuretic peptide, C-reactive protein, and creatinine clearance for prognosis in patients with known coronary heart disease.. Arch Intern Med.

[16] Stephens M, Scheet P (2005). Accounting for decay of linkage disequilibrium in haplotype inference and missing-data imputation.. Am J Hum Genet.

[17] Stephens M, Smith NJ, Donnelly P (2001). A new statistical method for haplotype reconstruction from population data.. Am J Hum Genet.

[18] Barrett JC, Fry B, Maller J, Daly MJ (2005). Haploview: analysis and visualization of LD and haplotype maps.. Bioinformatics.

[19] SAS Institute (2008). Statistical analysis software, release 9.2..

[20] Casas JP, Bautista LE, Smeeth L, Sharma P, Hingorani AD (2005). Homocysteine and stroke: evidence on a causal link from mendelian randomisation.. Lancet.

[21] R Foundation of Statistical Computing (2007). R v2.6.1..

[22] Wootton PT, Drenos F, Cooper JA, Thompson SR, Stephens JW (2006). Tagging-SNP haplotype analysis of the secretory PLA2IIa gene PLA2G2A shows strong association with serum levels of sPLA2IIa: results from the UDACS study.. Hum Mol Genet.

[23] Zacho J, Tybjaerg-Hansen A, Jensen JS, Grande P, Sillesen H (2008). Genetically elevated C-reactive protein and ischemic vascular disease.. N Engl J Med.

[24] Elliott P, Chambers JC, Zhang W, Clarke R, Hopewell JC (2009). Genetic Loci associated with C-reactive protein levels and risk of coronary heart disease.. JAMA.

[25] Casas JP, Shah T, Cooper J, Hawe E, McMahon AD (2006). Insight into the nature of the CRP-coronary event association using Mendelian randomization.. Int J Epidemiol.

[26] Brunner EJ, Kivimaki M, Witte DR, Lawlor DA, Davey Smith G (2008). Inflammation, insulin resistance, and diabetes--Mendelian randomization using CRP haplotypes points upstream.. PLoS Med.

[27] Lee M, Hong KS, Chang SC, Saver JL (2010). Efficacy of homocysteine-lowering therapy with folic Acid in stroke prevention: a meta-analysis.. Stroke.

[28] Schunkert H, Samani NJ (2008). Elevated C-reactive protein in atherosclerosis--chicken or egg?. N Engl J Med.

